# A Facile Interfacial Self-Assembly of Crystalline Colloidal Monolayers by Tension Gradient

**DOI:** 10.3390/mi9060297

**Published:** 2018-06-13

**Authors:** Dong Feng, Ding Weng, Jiadao Wang

**Affiliations:** State Key Laboratory of Tribology, Tsinghua University, Beijing 100084, China; fd14@mails.tsinghua.edu.cn (D.F.); dingweng@mail.tsinghua.edu.cn (D.W.)

**Keywords:** self-assembly, air-liquid interface, tension gradient, Marangoni convection, nanoparticle, monolayer

## Abstract

Many self-assembly approaches of colloidal monolayers have flourished but with some shortages, such as complexity, time-consumption, parameter sensitivity, and high-cost. This paper presents a facile, rapid, well-controlled, and low-cost method to prepare monolayers by directly adding silica particle suspensions containing water and ethanol to different liquids. A detailed analysis of the self-assembly process was conducted. The particles dove into water firstly, then moved up under the effect of the buoyancy and the tension gradient. The tension gradient induced the Marangoni convection and the relative motion between the water and the particles. At last, the particles were adsorbed at the air-water interface to minimize the free energy. The quality of the monolayers depended on the addition of sodium dodecyl sulfonate or ethanol in the water subphase. An interfacial polymerization of ethyl 2-cyanoacrylate was used to determine the contact angles of the particles at different subphase surfaces. The value of the detachment energy was positively associated with the contact angle and the surface tension. When the detachment energy decreased to a certain value, some particles detached from the surface, leading to the formation of a quasi-double layer. We also observed that the content of ethanol in suspensions influenced the arrangement of particles.

## 1. Introduction

Two-dimensional ordered arrangements of colloidal particles, commonly referred to as crystalline colloidal monolayers, have a wide range of applications in surface-enhanced Raman scattering (SERS) [1], patterned surface fabrication [2], photonic crystals [3], wetting property modification [4] and so on. Recently, numerous methods have been developed to prepare crystalline monolayers on various substrates. The main approaches are based on (1) the wettability of colloidal suspensions on the substrates or (2) the wettability of colloidal particles at the air-liquid interfaces. In the first case, the methods include spin coating [5], dip coating [6], and convective coating [7]. The suspensions form wetting films on the substrates and the order of the monolayers depends on the evaporation process of the suspensions. Thus, these methods are sensitive to small variations of ambient humidity or temperature, and careful adjustments of the experimental parameters have to be carried out [8]. Most predominantly, it is difficult to rearrange particles once they contact the substrates, thus resulting in defects. As for the second case, particles with some extent hydrophobicity float at the air-liquid interface, which provides the required particle mobility for defect-free packing. These processes generally consist of floating particles at the interfaces, crystallizing the particle films with a mobile barrier (Langmuir–Blodgett technique) [9] or a surfactant [10], and transferring the films onto substrates. Many methods are used to float the particles at the interfaces. One method consists of dispersing the particles in an organic liquid and slowly pipetting them onto the water surface. A monolayer formed at the water surface after evaporation of the solvent, which was time consuming [11]. Retsch et al. [12] deposited sparsely distributed particles on a parent substrate, followed by an immersion step, during which the particles detached from the substrate and floated on water. This process was a little complex and hard to control. Other methods require the formation of the meniscuses to enable the particles spread on the water surfaces. Zhang and co-workers [13] reported on the fabrication of 2D arrays of colloidal particles by a needle tip flow method. The position of the needle tip was controlled carefully to enable the spreading of the particles along the meniscus formed between the tip and the water surface. Another method was adding water to a level where a meniscus was formed around the periphery of a glass slide in a Petri dish, followed by dropping colloidal suspensions on the glass slide. Once the suspensions contacted water, the particles spread at the water surface [14]. In these methods, the precise control was needed to form the meniscus. Also, the supply of the particles should be below a certain value to prevent breaking of the water surface [15] and the efficiency was limited.

In this paper, we developed a facile, fast, and cost-effective method for the fabrication of large-area crystalline colloidal monolayers. SiO_2_ particles dispersed in a mixture of water and ethanol were directly added to the liquid. The monolayers could still form even though the suspensions dove into the liquid with high speed and big impact. They were subsequently transferred to a target substrate. Particle image velocimetry (PIV) was used to observe the moving-up of particles after the suspensions were added to the water, the mechanism of which was attributed to the buoyancy and the tension gradient. Scanning electron microscope (SEM) images of transferred monolayers indicated that the concentration variation of sodium dodecyl sulfonate (SDS) or ethanol in the water subphase influenced the order of the monolayers. The contact angles of SiO_2_ at different subphase surfaces were determined by a trapping technique. Then the influence of the wettability of particles on the formation of the monolayer was discussed. In previous studies, ethanol in the suspension was commonly thought to play a role as a spreading agent [16,17]. We found that the arrangement of particles was affected by the content of ethanol in the suspension.

## 2. Materials and Methods

### 2.1. Materials

The hydrophobic silica particle suspensions (2.5% *w/v*) with diameter of 900 nm were purchased from Tianjin Baseline Chromtech Research Center (Tianjin, China). The functional group on the surfaces of the particles was vinyl. The solvents were the mixtures of water and ethanol (1:1, *v/v*). Silicon (100) wafers (Zhejiang Lijing Silicon Material Co. Ltd., Quzhou, China) were cut into 1.5 × 1.5 cm^2^ and cleaned ultrasonically in acetone, ethanol, and deionized water for 20 min each in series. Acetone and ethanol were obtained from Beijing Chemical Works (Beijing, China). SDS was from J&K Scientific Ltd. (Beijing, China). The instant adhesive MC100 was produced by the 3M Company (Bracknell, UK). All other chemicals were of analytical grade and were used as purchased.

### 2.2. Monolayer Preparation

**Figure 1:**
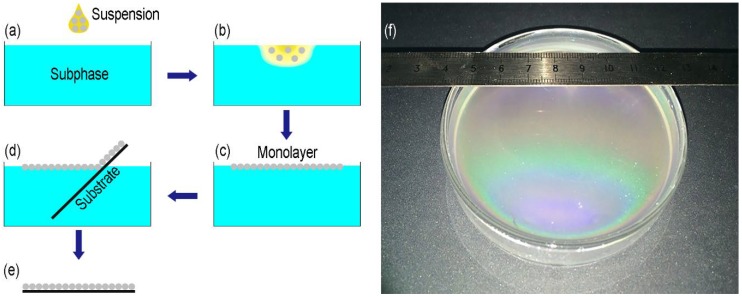
Schematic illustration of the monolayer fabrication process. (**a**) Addition of the hydrophobic silica suspension to the liquid; (**b**) Moving up of the particles to the air-liquid interface; (**c**) Self-assembly of a close-packed silica particle monolayer; (**d**) Pick-up of the monolayer with a silicon wafer; (**e**) Drying of the monolayer; (**f**) Photograph of the monolayer obtained from 900 nm particles.

Figure 1a–e shows the process flow for the self-assembly of the monolayer at the air-liquid interface and the subsequent transfer to a silicon wafer. The hydrophobic silica suspensions were directly added to the liquid (Figure 1a). The suspensions dove into the liquid and then the particles moved up to the air-liquid interface (Figure 1b). Patches of monolayers formed and floated at the interface (Figure 1c). With the further additions of the suspensions, the patches continued to grow in size until the whole interface was covered. The monolayer could be seen by the bright, colorful Bragg reflexes (>63 cm^2^, Figure 1f). A silicon wafer was immersed into the subphase and elevated under a shallow angle to transfer the silica monolayer (Figure 1d). Drying was performed under a dip angle of approximately 45° (Figure 1e). As a result, a hexagonally close-packed silica particle monolayer was obtained on the substrate. Even though the suspension was added at a height of 1.46 m above the water surface and parts of the suspension dove to a depth nearly 3 cm from the surface, the particles could still float up to form a monolayer (Video S1). Thus, the method was not sensitive to the dropping height. In the follow-up experiments, the dropping height was about 1 cm.

### 2.3. Determination of the Contact Angle of Nanoparticles at the Interface

**Figure 2:**
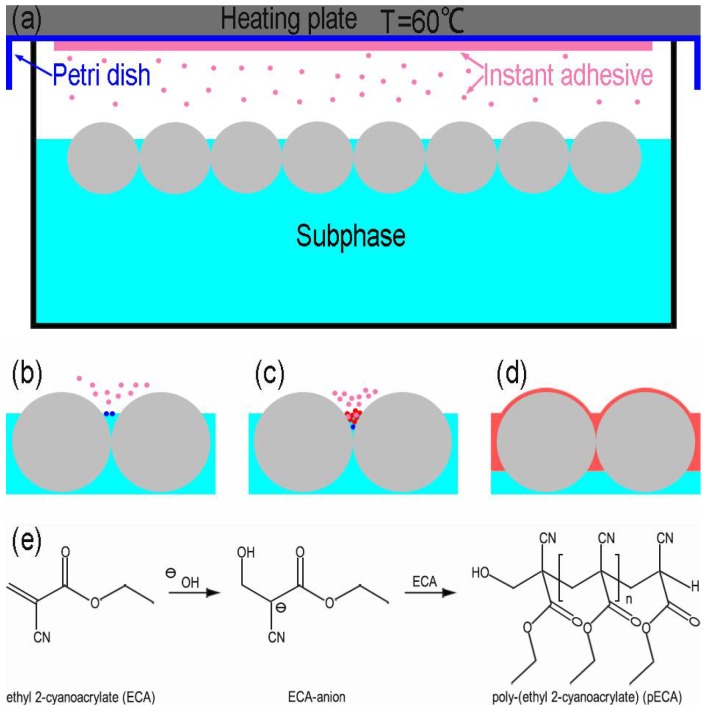
Interfacial polymerization of ethyl 2-cyanoacrylate (ECA) used to determine the contact angle of silica particles at the interface. (**a**) Scheme of the experimental setup; (**b**–**d**) Schematic of the reaction: The ECA monomers (pink) diffuse via the vapor phase to the air-liquid interface. The ECA anions (blue) are formed upon contact with the liquid. The anionic polymerization occurs and the polymer (red) is generated. The polymerization proceeds with the addition of monomers and the polymer membrane eventually covers the surfaces of the liquid and particles; (**e**) The polymerization reaction of ECA initiated by hydroxide ions in the liquid.

**Figure 3:**
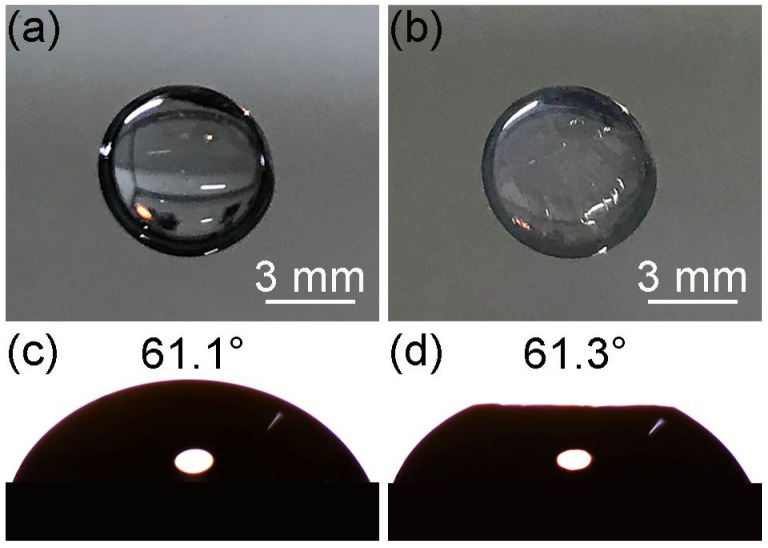
The appearance of a water drop on a silicon wafer before (**a**) and after (**b**) the polymer membrane forms on the drop surface; (**c**,**d**) are contact angle measurements of the water drop in (**a**,**b**), respectively.

**Figure 4:**
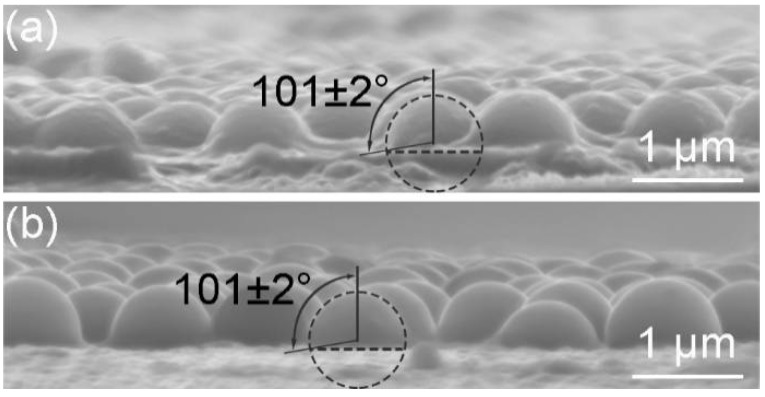
Side-view scanning electron microscope (SEM) images of the monolayers with the poly(ethyl 2-cyanoacrylate) (PECA), which are obtained at the deionized water surfaces. Different polymerization reaction times are used. (**a**) 5 min and (**b**) 20 min.

Inspired by the trapping technique based on the anionic polymerization reaction of butylcyanoacrylate [18], we used the instant adhesive MC100 (3M Company, Bracknell, UK) to visualize the interfacial position of silica particles. The main constituent of the adhesive was ethyl 2-cyanoacrylate (ECA) and the content of ECA was 90–95%. As shown in Figure 2a, a few drops of the instant adhesive were applied thinly and evenly to the bottom of a Petri dish. The Petri dish was upended on a weighing bottle containing a silica monolayer at the air-liquid interface and a closed space was formed. The instant adhesive was heated to 60 °C by a heating plate. The ECA monomers diffused through the vapor phase and contacted the liquid. The anions formed due to the reaction between the hydroxide ions (OH^−^) and the monomers, which initiated polymerization reaction (Figure 2b) [19]. As more monomers were supplied, a layer of poly(ethyl 2-cyanoacrylate) (PECA) appeared. The monomers could diffuse into the layer, leading to the growth of polymer into the subphase (Figure 2c). A thin polymer membrane formed around particles if there was a liquid film at the surfaces of particles (Figure 2d). Figure 2e shows the reaction scheme of the polymerization. After five minutes of reaction, the monolayer with PECA was transferred to a silicon wafer. In side-view images, the interfacial position of the particles could be visualized by SEM. This approach ensured that the monolayer and the interface were not perturbed. To illustrate that the polymerization process had no impact on the wettability, we performed contact angle measurement of a water drop (30 μL) on a silicon wafer. The contact angle was 61.1° (Figure 3c). Then the polymerization process occurred at the surface of the drop. Compared with Figure 3a, Figure 3b shows that a polymer membrane formed on the drop surface. The contact angle was 61.3° (Figure 3d). There was no significant change in the contact angle. This illustrated that the time required to form the membrane was so short that the wetting state of particles at the interface would not be influenced. The top of the drop became flat because the surface tension of water disappeared after the drop surface was covered by the membrane. In order to study whether the thickness of the polymer layer affected the contact angle of nanoparticles at the interface, we prolonged the polymerization reaction time to 20 min. The monolayer was obtained at the deionized water surface. Figure 4b shows that the membrane was much thicker than that when the time was 5 min in Figure 4a. The contact angle of the colloids did not change and the lateral arrangement of the colloids was not disturbed by the growing polymer film. Overall, the polymerization process was thought to possess little impact on the interfacial position of the silica particles.

### 2.4. Characterization

SEM (FEI Quanta 200 FEG, FEI Company, Hillsboro, OR, USA) was used to image the morphologies of the colloidal films. The motion of particles during the film formation process was recorded by PIV (MicroVec, Inc., Beijing, China) from a side view. The PIV setup included a 532 nm diode pump solid state laser (DPSSL) with a maximum power of 5 W and a 12–14 bit camera with a maximum capture rate of 258 fps, fitted with a 100 mm f/2.8 Tokina lens. When PIV was used to capture the motion of particles from above, the laser was replaced by a high brightness cold light source (XD-300, Nanjing Yanan Special Light Factory, Nanjing, China). The surface tension data were obtained using the hanging plate method with a Dataphysics OCA-20 (DataPhysics Instruments GmbH, Filderstadt, Germany) analyzer. Water contact angles were measured on a Dataphysics OCA 25 (DataPhysics Instruments GmbH, Filderstadt, Germany) instrument at room temperature.

## 3. Results and Discussion

### 3.1. Self-Assembly Process of the Colloidal Monolayer at the Air-Water Interface

**Figure 5:**
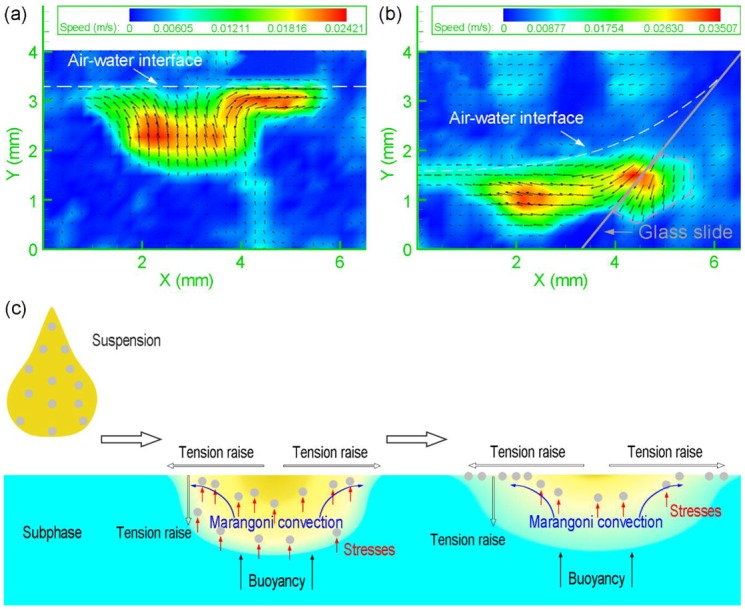
Transient motion of particles after the suspension is added to water (**a**) directly and (**b**) via a partially immersed glass slide at a tilt angle of approximately 45° with respect to the water surface. The direction of the arrows indicates the motion direction of the particles. The color scale bar shows the magnitude of the velocity (m/s). (**c**) Schematic illustration of the mechanism for particles moving up to the air-subphase interface.

**Figure 6:**
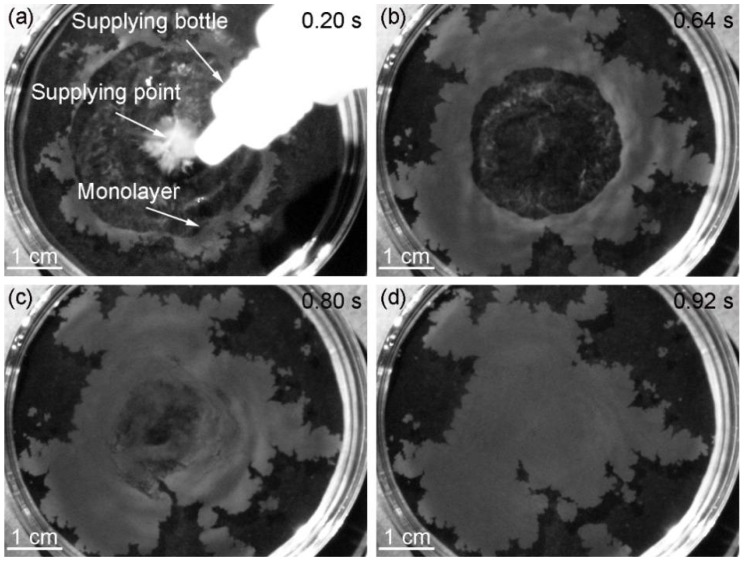
The appearance of the formation process of the monolayer at different time: (**a**) 0.20 s, (**b**) 0.64 s, (**c**) 0.80 s and (**d**) 0.92 s.

To clarify the formation mechanism of the monolayer, the motion of particles was recorded by PIV from a side view. The suspension drop dove into the water after it was added above the air-water interface. Then, the particles moved up to the air-water interface (Figure 5a). Another common method to produce monolayers was the usage of a partially immersed glass slide to add colloidal suspensions to the air–water interface, which was thought to be an effective approach to let the particles flow gently at the interface [20,21]. We used the above method to observe the motion of the particles after the suspension was injected onto the glass slide. It was found that some particles still dove into the water and then moved up to the interface (Figure 5b), which illustrated that the use of the glass slide would not have a significant impact on the quality of the monolayer. In the pink dashed line frame, no particles existed and the velocity distribution appeared because the glass slide reflected light. The reason why the particles could move up to the surface is attributed to three aspects (Figure 5c). Firstly, the suspension is under the effect of buoyancy due to the lower density of ethanol. Thus, the suspension does not sink into water. Secondly, a radiated concentration distribution of ethanol from high to low at the interface is formed when ethanol starts to dissolve into water, which is centered on the position where the suspension is dripped (the supplying point). Therefore, a radiated surface tension gradient from low to high is generated because the surface tension of ethanol is much lower than that of water. Variations in surface tension result in the Marangoni convection [22,23], which drives the particles to move up. Thirdly, the concentration of ethanol is higher at the position closer to the interface because of the convective transport and the dissolution process. The tension becomes lower and lower from the interior of subphase to the surface. The particles move up relative to water due to the stresses resulting from the variations of tension at the particle surfaces [24]. Without the relative motion, it is hard for the particles to move across and be absorbed at the air-water interface. In Figure 6, the motion of particles at the interface was recorded by PIV from a top view. The numerical values at the top right corners of the images represent the time of occurrence and the initial time was the moment when the drop of the suspension contacted the air-water interface. Silica particles moved radially under the effect of Marangoni convection and aggregated into a ring-like structure whose center was the supplying point (Figure 6a). As more particles moved to the interface, the ring became wide (Figure 6b). When the dissolution of ethanol reached the steady state, the surface tension gradient disappeared and the ring-like monolayer shrank to the supplying point (Figure 6c). In the end, the ring transformed into a piece of monolayer with a few small fragments around (Figure 6d). The time required for the whole process was about 0.92 s.

### 3.2. Influence of SDS Concentration in the Subphase on the Formation of the Monolayer

**Figure 7:**
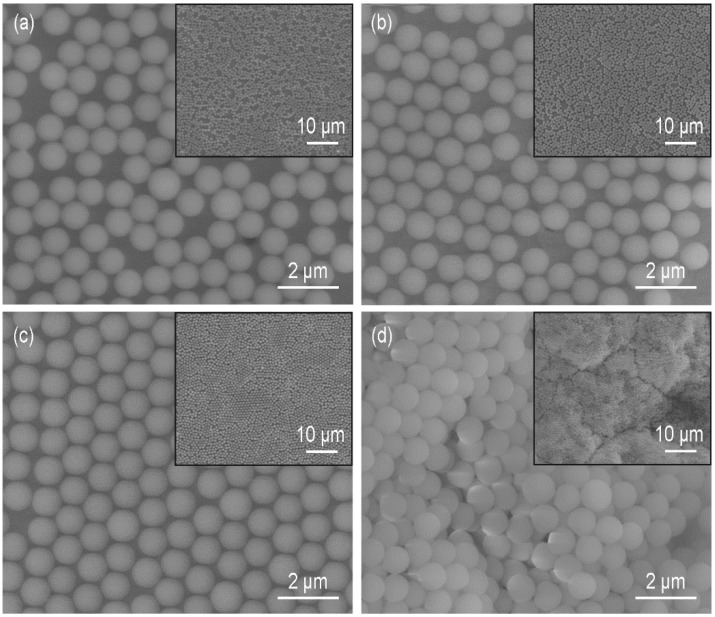
SEM images of monolayers formed at water surface with sodium dodecyl sulfonate (SDS) concentration of (**a**) 0; (**b**) 0.1; (**c**) 0.4 and (**d**) 0.7 mmol/L. The insets show lower magnifications of the same samples.

**Figure A1:**
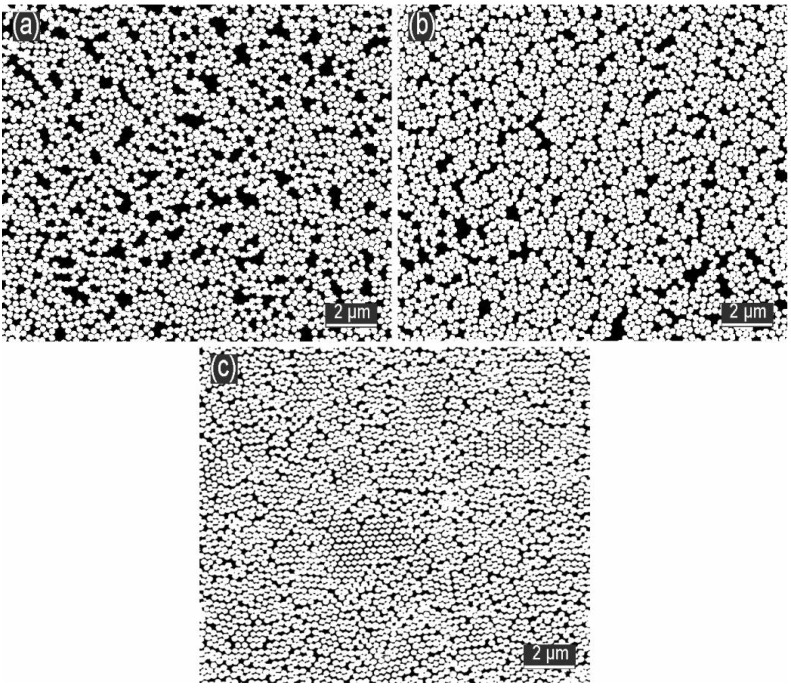
Treated SEM images of monolayers formed at water surface with SDS concentration of (**a**) 0; (**b**) 0.1 and (**c**) 0.4 mmol/L. The white parts are the monolayers and the black parts represent the substrates.

The research found that the quality of the monolayer was quiet dependent on the SDS concentration in the water subphase. The solvents of the silica suspensions were the mixtures of water and ethanol (1:1, *v/v*). The OTSU algorithm [25] was used to calculate the coverage rates of the monolayers on the silicon wafers (*φ*) in the insets of Figure 7a–c. In the treated pictures (Figure A1 in Appendix A), the monolayers and the substrates were divided into the white and black parts, respectively. By calculating the proportion of white pixels in the total pixels of the images, we got the coverage rates of the monolayers on the silicon wafers. For the hexagonally close-packed structure in an ideal state, *φ* is about 90.7% based on the geometrical calculation in Appendix B. Figure 7a shows a monolayer of silica particles formed at the deionized water surface. Most of the particles arranged in a disordered manner and *φ* was just 62.4%. The dominating attractive forces acting on the particles at the air-water interface are the van der Waals forces, the hydrophobic attractive forces, and the capillary forces [26]. Capillary forces are long range interactions with an effective range of up to several millimeters, while the ranges of hydrophobic forces and van der Waals forces are much shorter [26]. Particles brought into close vicinity by capillary forces immediately feel the hydrophobic forces and the van der Waals forces. The strong attractive forces cause the aggregation of particles and suppress the rearrangement of particles. When the SDS was added to the water prior to the spreading of the silica, the order and the coverage rate of the monolayer were greatly increased. As SDS molecules are amphiphilic, they accumulate at the air-water interface and act as a soft barrier. The particles rising to the interface push against the SDS molecules which in turn push the particles closer, therefore the coverage rate of the monolayer is increased. This phenomenon has been known as the piston oil effect [12]. Despite the equal sign of the charges, some SDS molecules are adsorbed at the surfaces of silica particles, which introduces more negative charges and enhances the electrostatic repulsion forces between particles [27]. The energy barrier for a close contact is increased. Moreover, SDS lowers the capillary forces by reducing the surface tension of water [28]. As a result, the repulsion forces could counteract the attractive forces, which enables particles to have sufficient mobility. The extrusion forces from the SDS arrange the particles into a crystalline monolayer. It is also found that the presence of surfactants makes the monolayer exhibit higher mechanical stability and less fracture at the edges. Thus, the addition of SDS has benefitted the monolayer transfer, too.

**Figure 8:**
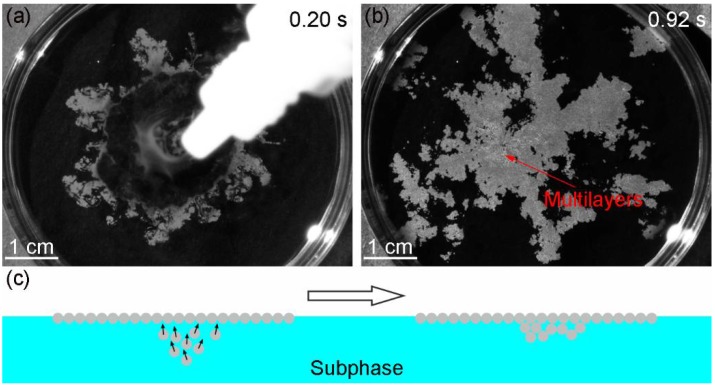
The appearance of the monolayer obtained with SDS concentration of 0.7 mmol/L at different time: (**a**) 0.20 s and (**b**) 0.92 s; (**c**) Schematic illustration of the formation mechanism of the multilayers. The solid arrows in (**c**) represent the motion direction of the particles.

The monolayers formed with SDS concentrations between 0.1 and 0.7 mmol/L are shown in Figure 7b–d. When the concentration of SDS was 0.1 mmol/L, some defects, such as vacancies, dislocations and disordered regions still existed, and *φ* increased to 66.8% (Figure 7b). At the most appropriate concentration (0.4 mmol/L), a crystalline monolayer with the highest coverage rate (*φ* = 78.7%) was obtained (Figure 7c). As the concentration reached 0.7 mmol/L, particles stacked into multilayers (Figure 7d). Figure 8a shows that the maximum inner diameter of the ring-like structure was much smaller than that when no SDS was added in Figure 6a, which illustrated that the amount of SDS at the air-water interface was high enough to impede the spreading of the particles. The multilayers (the whiter regions in Figure 8b) were mainly found near the supplying point. The formation mechanism is shown in Figure 8c. At the end of the self-assembly process, there are a small number of silica suspensions under the air-water interface. The Marangoni convection is too weak to overcome the counterforce of SDS. The monolayer shrinks and covers the air-water interface near the supplying point. When those silica particles move up, they could only move to the bottom of the monolayer and are fixed under the van der Waals forces between particles.

### 3.3. Influence of Ethanol Concentration in the Subphase on the Formation of the Monolayer

**Figure 9:**
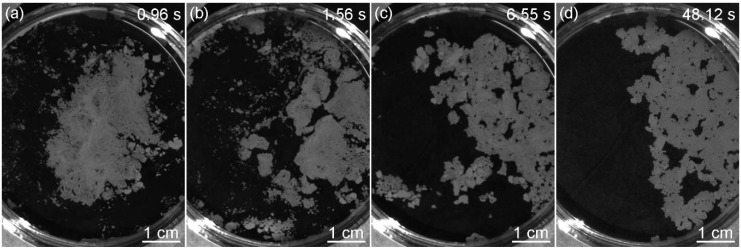
The appearance of the monolayer obtained with ethanol concentration of 20 vol.% at different time: (**a**) 0.96 s; (**b**) 1.56 s; (**c**) 6.55 s and (**d**) 48.12 s.

**Figure 10:**
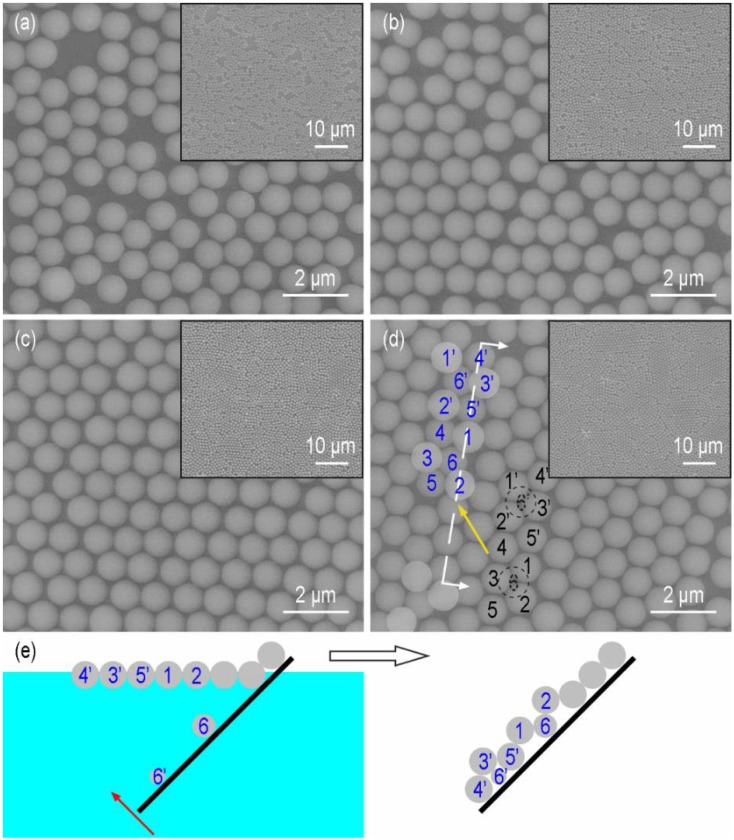
SEM images of monolayers formed at liquid surface with ethanol concentration of (**a**) 10; (**b**) 20; (**c**) 30 and (**d**) 40 vol.%. The insets show lower magnifications of the same samples. (**e**) Schematic illustration of the formation mechanism of the quasi-double layer, which is drew according to the cutting position (the white dashed line) and the projection direction (the white arrows) in (**d**).

**Figure 11:**
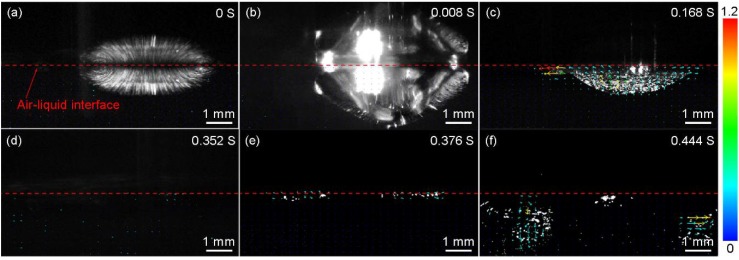
The movement process of particles after the suspension is added to the subphase containing 40 vol.% ethanol, which is captured by PIV. (**a**–**f**) correspond to the movements of particles at 0, 0.008, 0.168, 0.352, 0.376 and 0.444 s, respectively. The reflections above the interfaces are caused by the level fluctuations. The direction of the arrows indicates the motion direction of the particles. The color scale bar shows the magnitude of the velocity (cm/s).

**Figure A2:**
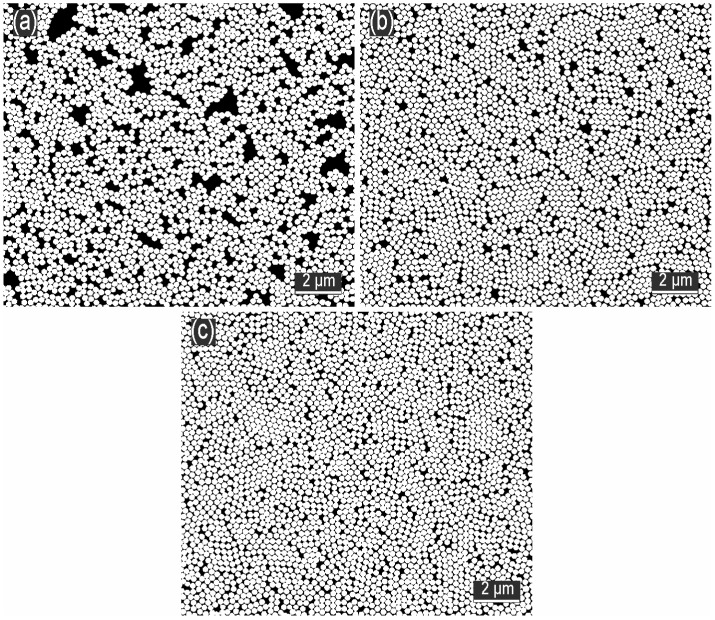
Treated SEM images of monolayers formed at liquid surface with ethanol concentration of (**a**) 10; (**b**) 20; (**c**) 30 vol.%. The white parts are the monolayers and the black parts represent the substrates.

Another parameter that could influence the formation of the monolayer was the ethanol concentration in the water subphase. The solvents of the silica suspensions were the mixtures of water and ethanol (1:1, *v/v*). With the increase in ethanol concentration in the subphase, the capillary forces decrease because the surface tension of the mixture becomes lower. The addition of ethanol could also decrease the hydrophobic attractive forces [29,30]. The regularity of the monolayer is improved as the attractive forces are counteracted more efficiently by the repulsive forces and the particles have more time to reach their minimum free energy position and crystalize into a hexagonally close-packed structure. Taking the case of ethanol concentration being 20 vol.%, the particles congregated when the Marangoni convection disappeared (Figure 9a). The monolayer was divided into pieces under the Marangoni convection induced by the ethanol evaporation near the container wall and then the pieces moved to the side where the convection was stronger (Figure 9b–d). This indicated the attractive forces between particles were weak, otherwise the monolayer would move as a whole. We also found that the slightly liquid sloshing on the surface led to the rearrangement of the particles, which was not observed without ethanol in the subphase. Thus, the addition of ethanol decreased the attractive forces between the particles. SEM images of monolayers formed at liquid surfaces with ethanol concentrations between 10 and 40 vol.% are shown in Figure 10. The treated insets of Figure 10a–c by the OTSU algorithm are shown in Figure A2 in Appendix A. When the ethanol concentration was 10 vol.%, the arrangement of particles was disordered (Figure 10a). The coverage rate *φ* was 65.7%, which was higher than that (*φ* = 62.4%) when no ethanol existed. As the ethanol concentration reached 20 vol.%, the order of the arrangement was improved and *φ* was 72.8% (Figure 10b). Figure 10c reveals that a monolayer with limited dislocations was obtained at the ethanol concentration of 30 vol.%. 74.6% of the substrate surface was covered by the silica monolayer. When the concentration of ethanol reached 40 vol.%, Figure 10d shows that there was a discretely distributed upper layer on the hexagonally ordered bottom layer, which formed a quasi-double layer. Each structural unit in the upper layer consisted of three particles arranged triangularly and every particle located in the interstice formed by three particles of the bottom layer. This kind of structure was also reported in previous papers [10,31,32]. The particles marked with black and blue characters made up the representative regions of the monolayer and the double layer, respectively. By analyzing the positional relationships between the particles of the double layer, the formation mechanism is similar with that in Reference [10]. At the concentration of 40 vol.%, some particles dive into the subphase after the addition of the suspension. In order to confirm this, we used PIV to observe the movements of particles from a side view after the suspension (1.5 × 10^−5^ *w/v*) was added to the subphase, as shown in Figure 11. The suspension was diluted in order to meet the requirement of PIV observation and the solvent remained unchanged. The initial time was the moment when the drop of the suspension contacted with the air-liquid interface (Figure 11a) and the particles moved downward with the suspension because of inertia (Figure 11b). Then, the particles moved up and approached the interface in Figure 11c. At 0.352 s, no particle was seen in Figure 11d, which illustrated the particles were at the interface. However, many particles were seen below the interface after 0.376 s in Figure 11e,f, which meant the particles could not be held at the interface. The reason is fully discussed in Section 3.4. During the transfer process, particles below the interface (6 and 6’ in Figure 10e) could be captured on the substrate and move to the bottoms of the interstices formed by every three neighboring particles in the monolayer. They push upper three particles 1, 2, 3 and 1’, 2’, 3’ (Figure 10d), respectively. Each of the particles 6 and 6’ comes into the monolayer and occupies the positions previously belonging to three particles. Particles 4, 5 and 4’, 5’ move towards particles 3 and 3’, respectively. The pentagonal structures are formed in the bottom layer.

### 3.4. Relationship Between the Particle Wettability and the Formation of the Monolayer

**Figure 12:**
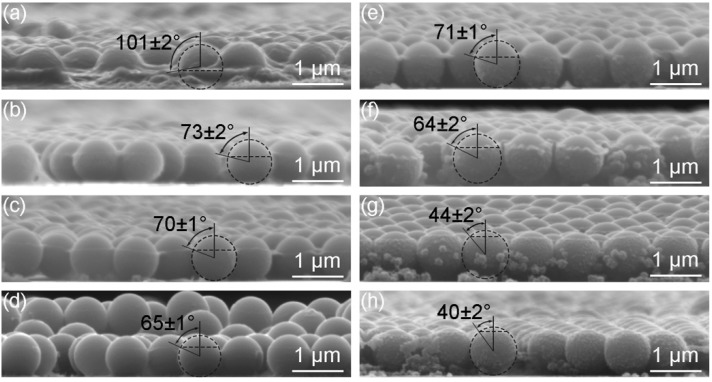
Side-view SEM images of the monolayers with the PECA. (**a**–**d**) correspond to monolayers obtained at SDS concentrations of 0, 0.1, 0.4, and 0.7 mmol/L; (**e**–**h**) correspond to monolayers obtained at ethanol concentrations of 10, 20, 30 and 40 vol.%. The data of θ are the average values of measurements made from five different particles.

The particle wettability can be properly described by the three-phase contact angle *θ*. Figure 12a–d show the side-view SEM images of monolayers obtained at the surfaces of subphases containing different concentrations of SDS. One could see horizontal boundaries representing the positions of subphase surfaces. PECA also formed around the parts of the particles below the boundaries, which reflected that the polymerization reaction could occur in the subphases. With no SDS in the subphase, the contact angle was 101°, which illustrated that the silica particles were hydrophobic (Figure 12a). The contact angle changed from 73° (Figure 12b) to 65° (Figure 12d) with the increase of the SDS concentration. Analogously, the contact angle went smaller with higher ethanol concentration as shown in Figure 12e–h. In other words, the particles were more immersed in the subphase. The surface tensions of different subphases are summarized in Table 1. It was worth mentioning that the surface tension of the subphase containing 0.4 mmol/L SDS was higher than that containing 10 vol.% ethanol, while the contact angles of particles at surfaces of these two subphases were similar. The tensions are related to the contact angle through Young’s equation [33]

(1) $$\gamma_{pa}=\gamma_{ps}+\gamma_{sa}\cos\theta$$

where *γ* is the appropriate interfacial tension and the subscripts *a*, *p*, *s* represent the air, particle and subphase, respectively; *θ* is the contact angle of a particle at a subphase surface. For different subphases, $\gamma_{pa}$ was equal. Thus, the value of $\gamma_{ps}$ of the subphase containing 0.4 mmol/L SDS was lower than that containing 10 vol.% ethanol. For the subphases containing 0.7 mmol/L SDS and 20 vol.% ethanol, the phenomenon was similar. One of the contribution factors is that the adsorption process of SDS changes the surface property of silica particles. The hydrophobic interactions between the alkyl chains of surfactants and the hydrophobic sites on the particles [34] lead to the conversion of particle surface state from the hydrophobicity to the hydrophilicity due to the ionic heads of the surfactants orienting towards the bulk solution. As mentioned above, some particles dove into the subphase when the ethanol concentration was up to 40 vol.%. The strength with which a particle is held at a subphase surface is related not only to *θ* but also to the surface tension $\gamma_{sa}$. Assuming the particle is small enough so that the effect of gravity is negligible and the subphase surface remains planar up to the contact line with the particle, the detachment energy E required to remove the particle from the surface into the subphase is given by [35]

(2) $$E=\pi R^{2}\gamma_{sa}{\left(1-\cos\theta \right)}^{2}$$

where *R* is the radius of the particle. The detachment energies of particles at different subphase surfaces are listed in Table 1. For the subphase containing 40 vol.% ethanol, the value of detachment energy ($2.65\times 10^{5}\;kT$) is much smaller than that ($160\times 10^{5}\;kT$) when no ethanol existed and the desorption of particles is the easiest. *k* is the Boltzmann constant and *T* is the temperature measured in Kelvin. However, it is considered that the particles are attached to the surface when the energy of adsorption is greater than the thermal energy *kT* [36]. Firstly, Equation (2) neglects the effect of the line tension, which is defined as the excess free energy per unit length of the line where the three phases meet. The detachment energies could be relatively low in the presence of the positive line tension [37]. The contribution of the line tension is important in the regime where the contact angle is larger than 120° or smaller than 60° [38]. Secondly, the impact of droplet on the surface when the suspension is added leads to the surface fluctuation, which is unfavorable to the adsorption of particles to the surface. Thirdly, the contact angle of interfacially absorbed particles has been found to possess a broad distribution [18,39]. Partial particles might have a contact angle lower than 40° and dive into the subphase. Although θ>90° is not necessary, the particles could not float at the surface as long as the detachment energy is too small. These experiments also shows that the formation of the multilayers when the subphase containing 0.7 mmol/L SDS (Figure 7d) is not due to the desorption of particles, because the detachment energy is much higher than that when the subphase containing 40 vol.% ethanol (Figure 10d).

### 3.5. Influence of Ethanol Concentration in the Suspension on the Formation of the Monolayer

**Figure 13:**
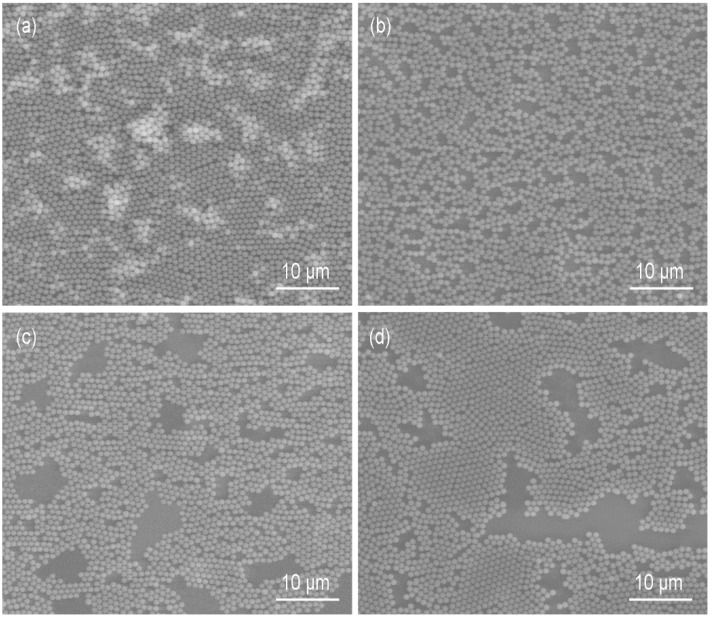
SEM images of monolayers formed by particles in different composition of ethanol/water: (**a**) 25%; (**b**) 50%; (**c**) 75% and (**d**) 100%.

**Figure 14:**
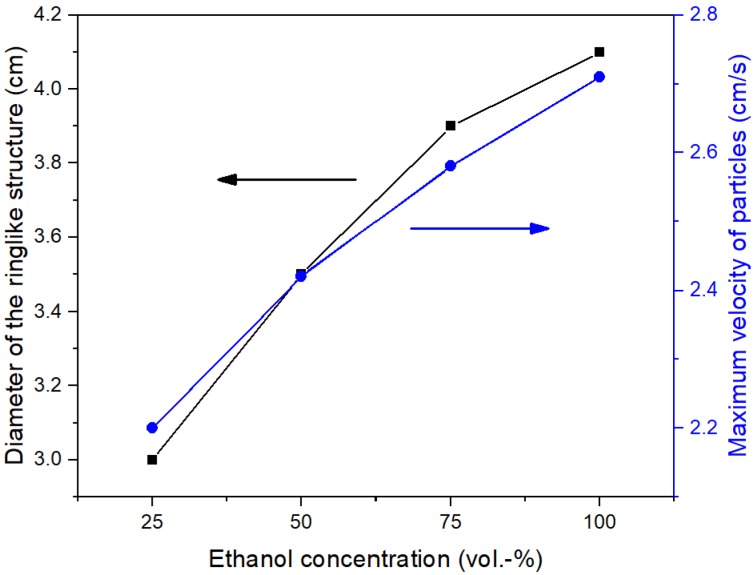
Influence of the ethanol concentration in the suspension on the maximum inner diameter of the ring-like structure (left-hand ordinate, ■) and on the maximum velocity of particles (right-hand ordinate, ●).

**Figure A3:**
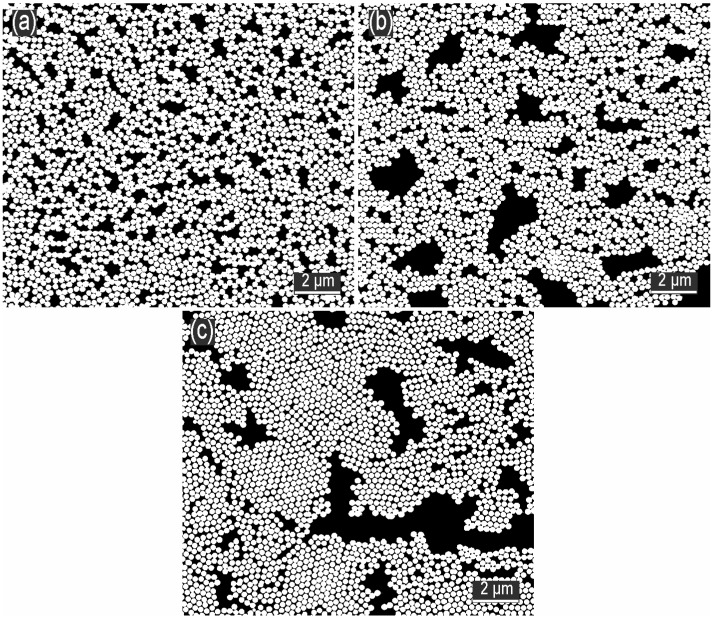
Treated SEM images of monolayers formed by particles in different composition of ethanol/water: (**a**) 50%; (**b**) 75% and (**c**) 100%. The white parts are the monolayers and the black parts represent the substrates.

We investigated the influence of ethanol concentration in the suspension on the formation of the monolayer by dispersing the particles in different compositions of ethanol/water while the weight percentage of particles in the mixture was fixed at 2.5%. The silica suspensions were added to the surfaces of deionized water. The treated insets of Figure 13b–d by the OTSU algorithm are shown in Figure A3 in Appendix A. When the volume percentage of ethanol was 25 vol.%, the particle film was a quasi-double layer structure consisting of a hexagonally close-packed bottom layer and a discretely distributed upper layer (Figure 13a). As mentioned above, the spreading of particles at the surface was outside-in due to the Marangoni convention. In Figure 14, the maximum velocity of particles induced by the low ethanol concentration was weak because the strength of the Marangoni convection depended on the surface tension gradient along the air-water interface [22]. Thus, the maximum inner diameter of the ring-like structure during the formation process of the monolayer decreased with the reduction of the ethanol concentration (Figure 14). At the ethanol concentration of 25 vol.%, the diameter was so small that there was not enough space for the subsequent particles to form into a monolayer. Figure 13b shows that there was enough ethanol for particles to spread into a monolayer at 50 vol.% concentration and *φ* was 62.4%. However, the arrangement of the monolayer was disordered because of the strong attractive forces acting at the particles. As the concentration reached 75 vol.%, *φ* decreased to 59.9% due to the further increase of the maximum inner diameter. Interestingly, the particles were arranged into more orderly structures (Figure 13c). Due to the low content of ethanol in one suspension drop, the influence of ethanol on the forces between particles is negligible. The important factor is the shearing stresses of the Marangoni convention exerted on the agglomerate. The increase of ethanol concentration improves the shearing forces, which could overcome the attractive forces. As a result, the particles in the agglomerate would change their positions to reduce the projected area against the interfacial flow and form into a hexagonally close-packed structure. This phenomenon is a kind of Kirkwood-Alder phase transition [40]. At the highest concentration, i.e., 100 vol.%, *φ* was only 57.1% (Figure 13d). Most importantly, the long range order was further improved which was even better than that in Figure 10c.

## 4. Conclusions

In summary, we have demonstrated a facile interfacial self-assembly procedure for the manufacture of large-area crystalline arrays of SiO_2_ particles. The SiO_2_ suspensions containing water and ethanol were directly added to water without other controls. The particles dove into water and subsequently moved up to the air-water interface attributed to the buoyancy and the tension gradient. On the one hand, the particles moved with the Marangoni convection of water resulting from the tension gradient. On the other hand, the upward motion of the particles relative to water appeared due to the tension gradient induced stresses. Once absorbed at the interface, particles formed a ring-like structure, which then transformed into a piece of monolayer. The optimum concentration of SDS (0.4 mmol/L) or ethanol (30 vol.%) in the water subphase helped SiO_2_ particles self-assemble into a hexagonally close-packed array. By measuring the three-phase contact angles of particles through an interfacial polymerization of ethyl 2-cyanoacrylate, the wettability of the particles at different subphase surfaces was confirmed to be different. When the subphase was deionized water, the contact angle (101°) was the biggest and the monolayer was disordered. As for the subphase containing 40 vol.% ethanol, the contact angle (40°) was the smallest and the quasi-double layer formed. The reason was that the detachment energy was just $2.65\times 10^{5}\;kT$ and the particles could not be steadily adsorbed at the air-liquid interface. Therefore, the appropriate wettability of particles was important to form crystalline monolayers. Moreover, the arrangements of the particles were affected by the ethanol concentrations in the suspensions, showing that the Marangoni convection played an important role in the self-assembly.

## Figures and Tables

**Table 1:** Contact angles and detachment energies of silica particles at surfaces of different subphases. The surface tensions of different subphases are listed.

Suphase	Surface Tension (mN/m)	Contact Angle (deg)	Detachment Energy ($\times 10^{5}\;kT$)
deionized water	72.98 ± 0.01	101 ± 2	160
0.1 mmol/L SDS	67.77 ± 0.03	73 ± 2	52.4
0.4 mmol/L SDS	60.24 ± 0.07	70 ± 1	40.3
0.7 mmol/L SDS	48.05 ± 0.01	65 ± 1	24.8
10 vol.% ethanol	51.19 ± 0.04	71 ± 1	36.0
20 vol.% ethanol	40.80 ± 0.02	64 ± 2	19.9
30 vol.% ethanol	35.30 ± 0.07	44 ± 2	4.30
40 vol.% ethanol	31.30 ± 0.05	40 ± 2	2.65

**Figure A4:**
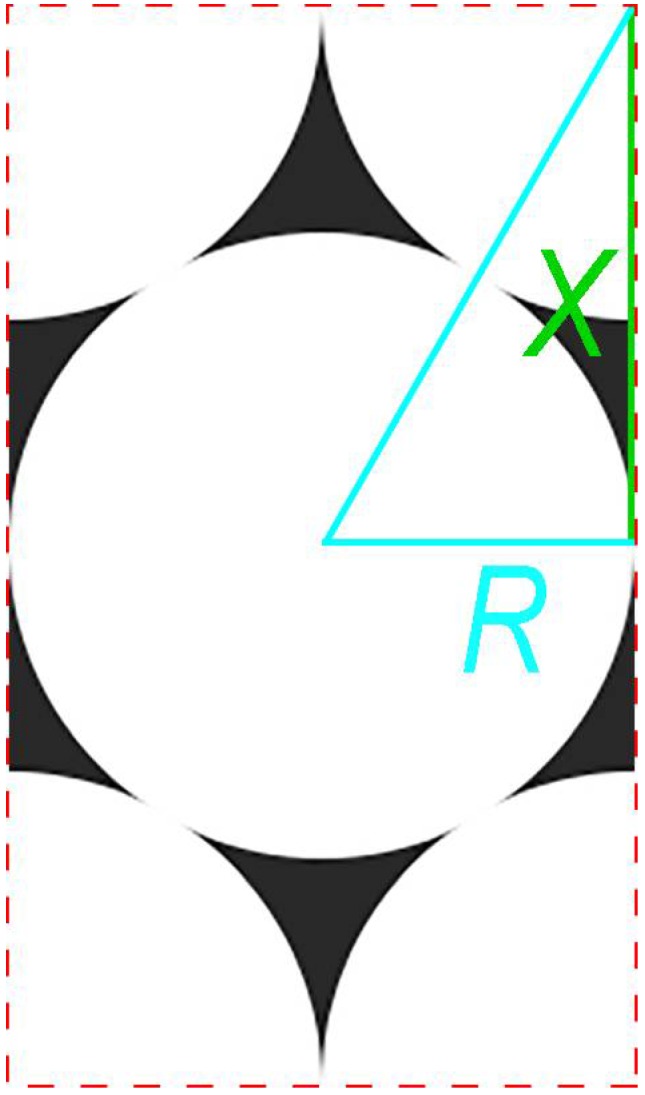
Structural unit of the ideal hexagonally close-packed structure.

## Supplementary Materials

The following video is available online at http://www.mdpi.com/2072-666X/9/6/297/s1, Video S1: The movement of particles after the addition of the suspension at a height of 1.46 m above the water surface.

## Appendix B

Figure A4 shows the structural unit of the ideal hexagonally close-packed structure, which could be used to calculate the coverage rate of the ideal monolayer on the silicon wafer.

Using Pythagoras, X can be calculated from the radius of the spheres as

(A1) $$X^{2}+R^{2}={\left(2R\right)}^{2}$$

(A2) $$X={\left(4R^{2}-R^{2}\right)}^{1/2}=\sqrt{3}R$$

The area S of the structural unit is given by

(A3) $$S=4RX=4\sqrt{3}R^{2}$$

The area $S_{0}$ of the particles in the structural unit is expressed as

(A4) $$S_{0}=2\pi R^{2}$$

Thus, the coverage rate φ of the ideal monolayer was

(A5) $$\varphi =\frac{S_{0}}{S}=\frac{\pi }{2\sqrt{3}}\approx 90.7\text{\%}$$
